# Transcriptional landscape of circulating platelets from patients with COVID-19 reveals key subnetworks and regulators underlying SARS-CoV-2 infection: implications for immunothrombosis

**DOI:** 10.1186/s13578-022-00750-5

**Published:** 2022-02-09

**Authors:** Weiping Ji, Lu Chen, Wei Yang, Ke Li, Jingting Zhao, Congcong Yan, Cancan You, Minghua Jiang, Meng Zhou, Xian Shen

**Affiliations:** 1 New Coronavirus Infectious Disease Prevention and Control Leadership Office, The Second Affiliated Hospital and Yuying Children’s Hospital of Wenzhou Medical University, Wenzhou, Zhejiang 325003 People’s Republic of China; 2 School of Biomedical Engineering, School of Ophthalmology & Optometry and Eye Hospital, Wenzhou Medical University, Wenzhou, Zhejiang 325027 People’s Republic of China; 3 School of Laboratory Medicine and Life Science, Wenzhou Medical University, 325035 Wenzhou, Zhejiang People’s Republic of China

**Keywords:** COVID-19, SARS-CoV-2, Platelets, Immunothrombosis, Transcriptome

## Abstract

**Background:**

Thrombosis and coagulopathy are pervasive pathological features of coronavirus disease 2019 (COVID-19), and thrombotic complications are a sign of severe COVID-19 disease and are associated with multiple organ failure and increased mortality. Platelets are essential cells that regulate hemostasis, thrombus formation and inflammation; however, the mechanism underlying the interaction between platelets and severe acute respiratory syndrome coronavirus 2 (SARS-CoV-2) remains unclear.

**Results:**

The present study performed RNA sequencing on the RNA isolated from platelets obtained from 10 COVID-19 patients and eight healthy donors, and discovered that SARS-CoV-2 not only significantly altered the coding and non-coding transcriptional landscape, but also altered the function of the platelets, promoted thrombus formation and affected energy metabolism of platelets. Integrative network biology analysis identified four key subnetworks and 16 risk regulators underlying SARS-CoV-2 infection, involved in coronavirus disease-COVID-19, platelet activation and immune response pathways. Furthermore, four risk genes (upstream binding transcription factor, RNA polymerase II, I and III subunit L, Y-box binding protein 1 and yippee like 2) were found to be associated with COVID-19 severity. Finally, a significant alteration in the von Willebrand factor/glycoprotein Ib-IX-V axis was revealed to be strongly associated with platelet aggregation and immunothrombosis.

**Conclusions:**

The transcriptional landscape and the identification of critical subnetworks and risk genes of platelets provided novel insights into the molecular mechanisms of immunothrombosis in COVID-19 progression, which may pave the way for the development of novel therapeutic strategies for preventing COVID-19-associated thrombosis and improving the clinical outcome of COVID-19 patients.

**Supplementary Information:**

The online version contains supplementary material available at 10.1186/s13578-022-00750-5.

## Background

Coronavirus disease 2019 (COVID-19) is caused by severe acute respiratory syndrome coronavirus 2 (SARS-CoV-2) [1] and represents one of the greatest public health challenges since the 1918 influenza pandemic over 100 years ago [2]. The main epidemiological characteristics of COVID-19 are rapid spread, high infectiousness and an approximately 1–3% mortality rate [3–6]. Due to the lack of specific treatment options available [7, 8], symptomatic treatment and supportive care remain the primary treatment strategy.

The respiratory symptoms associated with COVID-19 can lead to acute respiratory distress syndrome, in severe cases [9]. In addition, accumulating evidence has suggested that thrombosis and coagulopathy are pervasive pathological features of COVID-19 [10, 11]. Compared with other respiratory infectious diseases, COVID-19 shows a higher cumulative incidence of thrombotic complications [12–14], especially in COVID-19 patients admitted to intensive care units (ICUs) [15, 16]. It is noteworthy that hospitalized children with COVID-19 have been reported to be at an increased risk of developing thrombosis [17, 18]. In addition, the thrombotic complications of COVID-19 patients are associated with multiple organ failure and increased mortality [19, 20].

Autopsies of COVID-19 patients have shown that platelet-rich thrombi exist in the microcapillaries of multiple organs. The number of megakaryocytes in the heart and lungs is abnormally increased [21–23]. Previous studies have observed that the platelets of COVID-19 patients appear to be overreactive and may interact with SARS-CoV-2 to promote coagulation dysfunction during COVID-19 infection [24–26]. Platelets, the second most abundant type of cell in the peripheral blood [27], are best known as mediators of thrombus formation and hemostasis [28]. In recent years, studies have reported that platelets were the key sentinel and effector cells in infectious diseases, such as Dengue virus (DENV) and malaria [29, 30]. During pathogen invasion, the transcriptome and proteome of platelets have been reported to be altered to augment host defense mechanisms [31, 32]; however, these changes may also result in adverse outcomes. Activated platelets release various cytokines, including chemokines CXCL1, PF (platelet factor)-4, to amplify thrombin production, enhance leukocyte recruitment, promote neutrophil extracellular trap formation, upregulate the endothelial expression of proinflammatory cytokines and, finally, induce immunothrombosis [20]. Nevertheless, the mechanism underlying the interaction between platelets and SARS-CoV-2 remains unknown to the best of our knowledge.

The present study performed RNA sequencing (RNA-seq) on RNA isolated from platelets obtained from 10 COVID-19 patients and eight healthy donors and further examined the transcriptional dysregulation in circulating platelets from COVID-19 patients. Then, integrative network biology analysis was performed to identify critical subnetworks and risk regulators underlying SARS-CoV-2 infection. Our findings may provide novel insights into the molecular mechanisms of immunothrombosis in COVID-19 progression and pave the way for developing therapeutic strategies for preventing COVID-19-associated thrombosis and improving the clinical outcome of COVID-19 patients.

## Results

### Clinical characteristics of healthy donors and hospitalized patients with COVID-19

**Fig. 1:**
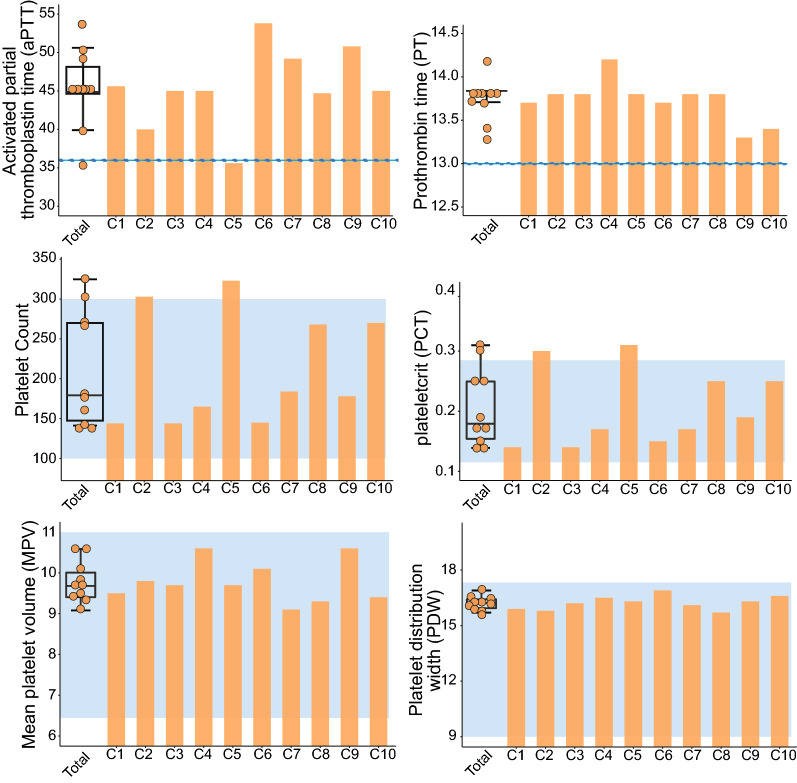
Laboratory parameters associated with hemostasis and coagulation in COVID-19 patients, including aPTT, PT, platelet count, PCT, MPV and PDW. COVID-19, coronavirus disease 2019; aPTT, activated partial thromboplastin time; PT, prothrombin time; PCT, plateletcrit; MPV, mean platelet volume; PDW, platelet distribution width

As shown in Table 1, COVID-19 patients were matched with healthy donors by age and sex. Comorbidities, including diabetes, hypertension and cancer, were present in 40% of the COVID-19 cases, which is consistent with previous reports [33–35]. The symptoms of the patient’s initial diagnosis mainly include fever (80%), cough (80%) and sputum (50%), occasionally sore throat (30%), fatigue (10%), muscle aches (20%), and nausea (10%). And the respiratory rate and heart rate of the hospitalized patients were in the normal range. Additionally, in Additional file 1: Fig. S1, the imaging of all patients was consistent with the changes of viral pneumonia. The CRP levels were significantly increased in the COVID-19 patient group, whereas the lymphocyte count was notably decreased, which is consistent with previous reports that found that elevated CRP levels and lymphopenia were the main laboratory characteristics of COVID-19 patients [36–39]. As shown in Fig. 1, the platelet count, plateletcrit (PCT), mean platelet volume (MPV), platelet distribution (PDW), prothrombin time (PT), and activated partial thromboplastin time (APTT) of the COVID-19 patient group were either within the normal ranges or only slightly exceeded the normal ranges, but the platelet count was substantially lower, and the PT and APTT were significantly increased compared with the control group. Furthermore, the alanine aminotransferase and creatinine levels between the COVID-19 patients and the healthy donors were not significantly different and were all within the normal range, indicating that liver and kidney damage or failure was not present in the COVID-19 patient cohort. The above results support that all patients enrolled in the group are ordinary new coronavirus patients.

**Table 1:** Clinical characteristics of control donors and hospitalized patients with COVID-19

	Control donors	COVID-19 patients	*P*-value
	(n = 8)	(n = 10)	
Age, years	47.25 (± 8.31)	45.80 (± 13.76)	0.797^a^
Gender, male	4 (50%)	6 (60%)	0.520^b^
*Comorbidities*			
Hypertension	0 (0%)	3 (30%)	
Diabetes	0 (0%)	2 (20%)	
Cancer	0 (0%)	1 (10%)	
Hyperlipidemia	3 (37.5%)	0 (0%)	
Heart disease	0 (0%)	0 (0%)	
DVT	0 (0%)	0 (0%)	
*Symptom*			
Fever	0 (0%)	8 (80%)	
Cough	0 (0%)	8 (80%)	
Expectoration	0 (0%)	5 (50%)	
Sore throat	0 (0%)	3 (30%)	
Fatigue	0 (0%)	1 (10%)	
Muscle ache	0 (0%)	2 (20%)	
Chest tightness	0 (0%)	0 (0%)	
Nausea	0 (0%)	1 (10%)	
Diarrhea	0 (0%)	0 (0%)	
*Physical signs*			
Respiratory rate		20 (18–20)	
Heart rate		85 (75–92)	
*Laboratory findings on admission*			
CRP, mg/dL	1.41 (± 1.78)	22.51 (± 25.65)	0.029^a^
Leukocytes, × 10^9^/L	5.39 (± 1.34)	4.85 (± 1.54)	0.440^a^
Neutrophils, × 10^9^/L	3.10 (± 0.85)	3.00 (± 1.33)	0.846^a^
Lymphocytes, × 10^9^/L	1.88 (± 0.66)	1.27 (± 0.28)	0.037^a^
Platelet count, × 10^9^/L	294.25 (± 44.52)	212.40 (± 70.70)	0.009^a^
MPV, fl	10.06 (± 0.81)	9.78 (± 0.51)	0.407^a^
PT, S	12.31 (± 0.75)	13.73 (± 0.25)	0.399^a^
APTT, S	36.40 (± 4.19)	45.47 (± 5.17)	0.001^a^
Fibrinogen, mg/Dl	2.86 (± 0.54)	4.76 (± 0.87)	< 0.001^a^
ALT, U/L	21.25 (± 10.47)	26.70 (± 5.76)	0.215^a^
Creatinine, μmol/L	60.34 (± 12.74)	66.75 (± 21.00)	0.440^a^

DVT, deep vein thrombosis; CRP, C reactive protein; MPV, mean platelet volume; PT, prothrombin time; APTT, activated partial thromboplastin time; ALT, alanine aminotransferase

^a^ Two-tailed Student T test

^b^ Chi-square test

### SARS-CoV-2 infection alters the coding and non-coding transcriptional landscape of human platelets

**Fig. 2:**
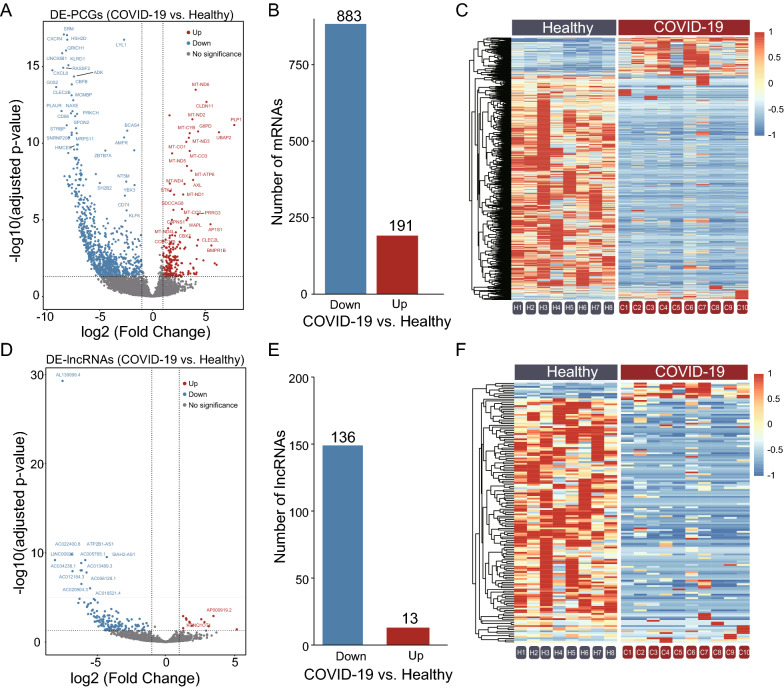
SARS-CoV-2 infection alters the coding and non-coding transcriptional landscape of human platelets. We performed RNA-seq on RNA isolated from highly purified platelets obtained from 10 COVID-19 patients and eight healthy donors. **A** Volcano plot depicting log_2_FC and FDR-adjusted P-values comparing COVID-19 and control samples. Differentially expressed up- (red) and downregulated (blue) PCGs are shown and selected PCGs are highlighted. **B** Bar plot with the number of significantly upregulated (red) and downregulated (blue) PCGs in COVID-19 patients compared with control samples. **C** Hierarchical clustering map of PCGs between COVID-19 patients and control samples. **D** Volcano plot depicting log_2_ transformed FC and FDR-adjusted P-values between COVID-19 and control samples. Differentially expressed up- (red) and downregulated (blue) lncRNAs are shown and selected lncRNAs are highlighted. **E** Number of significantly upregulated (red) and downregulated (blue) lncRNAs in COVID-19 patients compared with control samples. **F** Hierarchical clustering map of lncRNAs between COVID-19 patients and control samples. COVID-19, coronavirus disease 2019; PCGs, protein-coding genes; lncRNAs, long non-coding RNAs; FC, fold change; FDR, false discovery rate; RNA-seq, RNA sequencing

To determine whether SARS-CoV-2 infection altered the transcriptome of circulating human platelets, we performed RNA-seq on RNA isolated from platelets obtained from 10 COVID-19 patients and eight healthy donors. Hierarchical clustering of transcriptome-wide RNA expression showed differential grouping of COVID-19 patients and healthy donors (Additional file 2: Figure S2), suggesting that SARS-CoV-2 infection altered the transcriptional landscape of platelets. Differential expression analysis identified 1,223 differentially expressed RNAs between COVID-19 patients and healthy donors (adjusted P-value < 0.05 and absolute log_2_FC > 1.0), including 191 and 13 significantly upregulated mRNAs and lncRNAs, respectively, and 883 and 136 significantly downregulated mRNAs and lncRNAs, respectively (Fig. 2 and Additional file 5: Table S1).

**Fig. 3:**
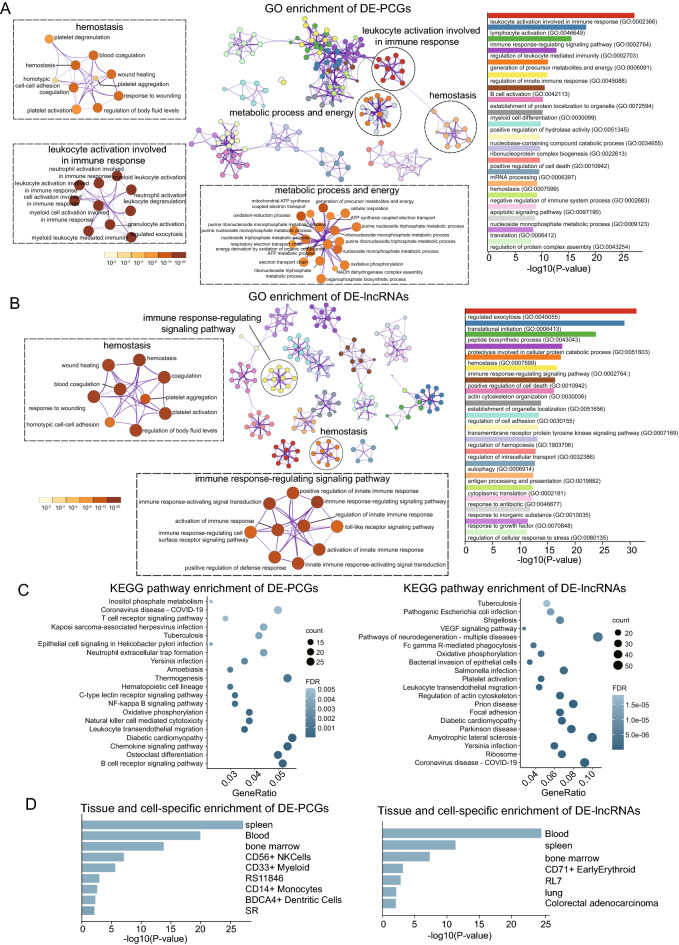
Functional enrichment analyses of differentially expressed RNAs. Significantly enriched GO functional terms associated with differentially expressed **A** mRNAs and **B** lncRNAs were identified using Metascape. The color of the subnetwork represents the P-value and the bar graph’s color represents each class of GO term. **C** The top 20 most enriched KEGG signaling pathways for the differentially expressed mRNAs and lncRNAs. The size and color of the circle represent the enriched count and P-value, respectively. **D** Bar plot shows the tissue and cell-specific enrichment result. lncRNAs, long non-coding RNAs; GO, Gene Ontology; KEGG, Kyoto Encyclopedia of Genes and Genomes

We performed GO functional term and KEGG signaling pathway enrichment analyses to determine the involvement of differentially expressed RNAs in biological processes. GO functional enrichment analysis revealed that differentially expressed mRNAs and lncRNAs were significantly enriched in biological processes involved in ‘hemostasis’, ‘platelet activation’, ‘immune response’ and ‘metabolic process and energy’ (Fig. 3A, B). The top 20 enriched KEGG signaling pathways are shown in Fig. 3C, in which differentially expressed mRNAs and lncRNAs were highly clustered in several signaling pathways associated with ‘coronavirus disease-COVID-19’, ‘platelet activation’ and ‘immune’. In addition, differentially expressed mRNAs and lncRNAs were observed to be preferentially expressed in the blood, spleen and bone marrow, as determined using tissue and cell-specific enrichment analysis (Fig. 3D). These data suggested that SARS-CoV-2 infection may alter the expression of platelet RNAs, change the function of platelets, promote thrombosis and affect the biological processes of energy metabolism.

### Integrative network biology analysis of circulating platelets from COVID-19 patients

**Fig. 4:**
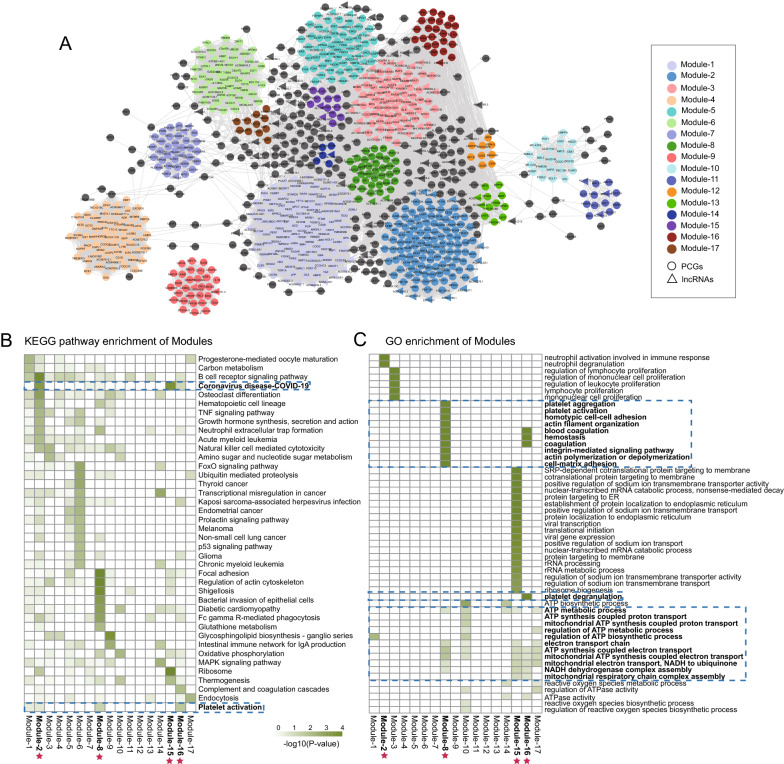
Identification and functional characterization of key subnetworks in circulating platelets during SARS-CoV-2 infection. **A** Global dysGCN in circulating platelets during SARS-CoV-2 infection. Network modules identified through the MFOLD are shown in distinct colors. Highly connected hub genes are labeled with respective gene symbols. **B** Heat map of enriched KEGG signaling pathways with FDR-adjusted P-value < 0.01 among different network modules. **C** Heat map of enriched GO functional terms with FDR-adjusted P-value < 0.01 among different network modules. dysGCN, dysregulated gene co-expression network; SARS-CoV-2, severe acute respiratory syndrome coronavirus 2; GO, Gene Ontology; KEGG, Kyoto Encyclopedia of Genes and Genomes; FDR, false discovery rate

To gain further insight into the biological network of transcriptional changes associated with SARS-CoV-2 infection and platelet activation, we constructed a dysGCN based on differentially expressed mRNAs and lncRNAs by calculating the PCC. The dysGCN was built and included 960 nodes (118 lncRNAs and 842 mRNAs) and 30,716 connections between them (Fig. 4A). After module mining, we identified a total of 17 co-expression modules ranging in size from 6 to 130 RNAs. Functional characterization using GO functional term and KEGG signaling pathway enrichment analyses showed that modules-2, -8 and -16 were enriched in ‘coronavirus disease-COVID-19’ and ‘platelet activation pathways’. In addition, module-2 was also associated with ‘immune response pathways’ and module-15 was enriched in the ‘coronavirus disease-COVID-19 pathway’ only (Fig. 4B, C). These results suggested that modules-2, -8, -15 and -16 are key subnetworks involved in circulating platelets during SARS-CoV-2 infection.

### Systematic identification of key regulators in circulating platelets during SARS-CoV-2 infection

**Fig. 5:**
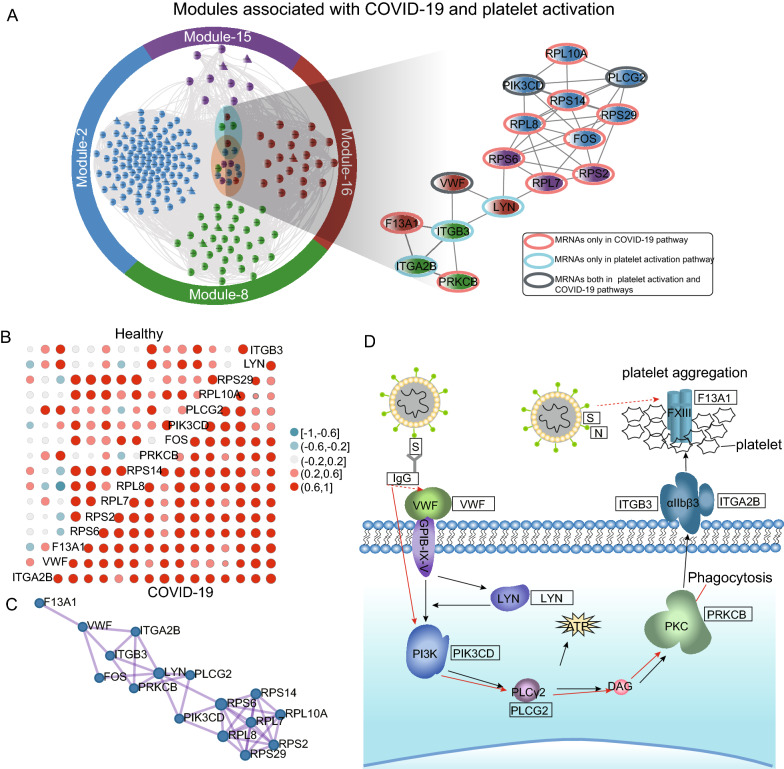
Identification of subnetwork and core genes associated with SARS-CoV-2 infection and platelet activation. **A** The subnetwork associated with SARS-CoV-2 infection and platelet activation in modules-2, -8, -15 and -16. The red round border indicates mRNAs only in the ‘COVID-19 pathway’; blue round border indicates mRNAs only in ‘platelet activation pathway’; gray round border indicates mRNAs both in ‘platelet activation’ and ‘COVID-19’ pathways. **B** Pearson’s correlation analysis was estimated among the core mRNAs. The size of the circle scales with the correlation magnitude and the darker the color, the larger the magnitude of the correlation coefficient. Red and blue represent positive and negative correlations, respectively. The upper triangular matrix and the lower triangular matrix represent the correlation coefficients in healthy and COVID-19 samples, respectively. **C** Protein–protein interaction network analysis among the core mRNAs. **D** Schematic diagram illustrating the SARS-CoV-2-induced activation of platelets and subsequent enhanced occurrence of thrombosis in COVID-19 patients. The red and black arrows indicate the pathways of SARS-CoV-2 infection and platelet activation, respectively. The dashed line indicates the indirect action and the solid line indicates the direct action. SARS-CoV-2, severe acute respiratory syndrome coronavirus 2; COVID-19, coronavirus disease 2019

The four modules formed a key subnetwork, consisting of 196 nodes (17 lncRNAs and 179 mRNAs) and 8,822 edges between them, and were mainly involved in the biological process and RNA interaction of platelet activation caused by SARS-CoV-2 infection. To discover critical regulators of this subnetwork during SARS-CoV-2 infection, we mapped these mRNAs to ‘coronavirus disease-COVID-19’ and ‘platelet activation pathways’, and identified 16 key candidate regulators (Fig. 5A). These 16 key candidate regulators were predominantly expressed in the blood and spleen, as shown in tissue and cell-specific enrichment analysis (Additional file 3: Figure S3A). Further analysis using disease and viral perturbation datasets from the GEO database also showed that these key candidate regulators were associated with blood coagulation-related diseases (Additional file 3: Figure S3B) and were differentially expressed in SARS-CoV-2- or SARS virus-associated GEO datasets (Additional file 3: Figure S3C).

Furthermore, we calculated the expression correlation of these 16 key candidate regulators in healthy donors and disease samples. As shown in Fig. 5B, the expression levels of these 16 key candidate regulators were significantly correlated in COVID-19 patients, which were not observed in healthy donors. These observations indicated that these regulators were differentially expressed and exhibited a highly synergistic pattern during SARS-CoV-2 infection. We observed a significant overlap between the co-expression relationship and protein–protein interactions (PPIs) among the 16 key candidate regulators when considering the physical interaction.

Finally, we combined the results obtained from the enrichment analyses, identification of key subnetworks and regulators, PPI network and previous studies to infer the key active pathway involved in platelet aggregation during SARS-CoV-2 infection. As shown in Fig. 5D, von Willebrand factor (VWF) bound to the platelet membrane glycoprotein (GP) Ib-IX-V complex and initiated a signaling cascade, which subsequently activated LYN proto-oncogene, Src family tyrosine kinase (LYN). The downstream pathway was initiated by LYN and propagated through PI3K, phospholipase C γ2 and protein kinase C, which promoted ATP secretion and finally caused αIIbβ3-mediated platelet activation and aggregation.

We measured the levels of four DEmRNAs (F13A1, ITGB3, ITGA2B and VWF) involved in platelet aggregation during SARS-CoV-2 infection in 5 patients with pneumonia and 6 normal donors by RT-qPCR. As shown in Additional file 4: Figure S4, we found that the ITGA2B and VWF were significantly decreased in patients with pneumonia compared with control donors. In addition, the F13A1 and ITGB3 also showed lower expression levels in patients with pneumonia compared with control donors although the difference did not reach statistical significance which might be due to the limitation of sample size. These RT-qPCR results are in line with our observation form RNA-seq.

### Identification of risk regulators associated with COVID-19 severity

**Fig. 6:**
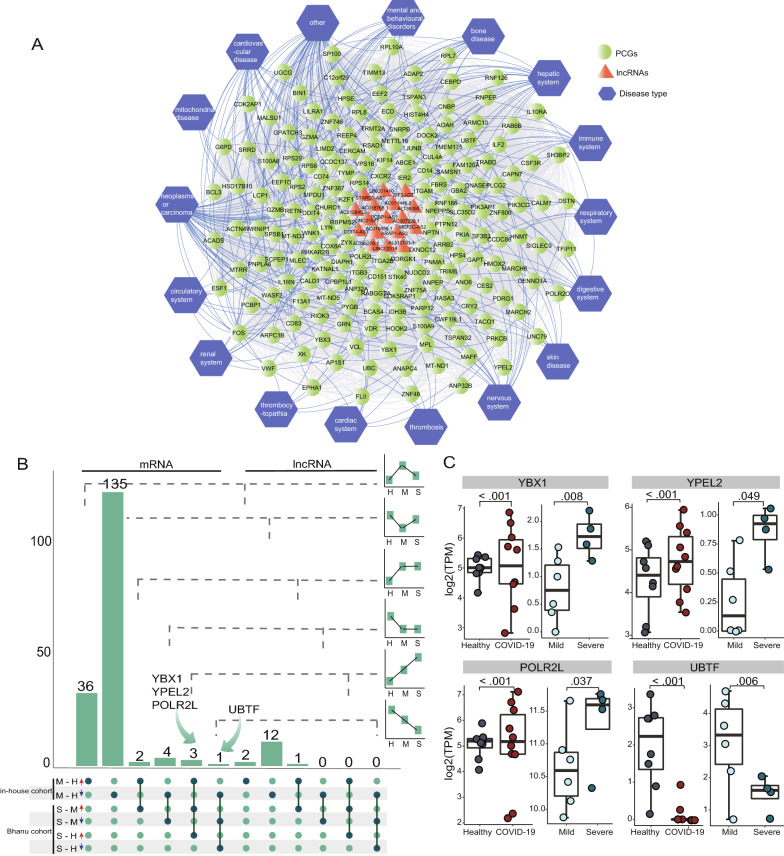
Differentially expressed RNAs are closely associated with disease progression. **A** Network association between diseases and differentially expressed RNAs. The highlighted blue color node represents the disease associated with the RNAs, the green node represents the differentially expressed mRNAs and the red node represents the differentially expressed lncRNAs. **B** UpSet plot shows the number of differentially expressed RNAs in the subnetwork with six expression patterns in M—H and S—M comparisons. The bar and line charts show the trend of each group, and the arrows outline the position of the candidate mRNA. **C** Consistently downregulated or upregulated mRNA expression. Gray dots indicate healthy donors in the present study cohort; red dots indicate mild patients in the present study cohort; light green dots indicate mild patients in Bhanu’s cohort; and deep green dots indicate severe patients in Bhanu’s cohort. M, mild; H, health; S, severe; lncRNAs, long non-coding RNAs

COVID-19 patients are often accompanied by many complications, and the development of these complications often contributes to the progression of the disease and even death [40, 41]. We used RNAs identified in the key subnetwork to construct an RNA-disease association network to investigate how COVID-19-associated transcriptional characteristics of circulating platelets were shared with other diseases. Information related to disorders, genes, and possible association was obtained from the Online Mendelian Inheritance in Man (OMIM) and CTD databases. We classified the associated disorders into 19 categories. As shown in Fig. 6A, the number of interconnected genes with diseases suggested that key subnetworks in circulating platelets underlying SARS-CoV-2 infection were closely associated with immunological, cardiovascular, liver and hematological diseases, and cancer.

To further examine whether RNAs in the key subnetwork were risk regulators associated with COVID-19 severity, we compared the expression levels of RNAs in the key subnetwork between non-ICU COVID-19 patients and healthy individuals [mild (M)—health (H)], ICU COVID-19 patients and healthy individuals [severe (S)—H], and ICU COVID-19 patients and non-ICU COVID-19 patients (S—M) in both our and Bhanu’s cohorts. According to their expression levels, we identified four RNAs as candidate risk regulators associated with COVID-19 severity (Fig. 6B). As shown in Fig. 6C, RNA polymerase II, I and III subunit L (POLR2L), Y-box binding protein 1 (YBX1) and yippee like 2 (YPEL2) were revealed to be upregulated, while upstream binding transcription factor (UBTF) was downregulated.

### COVID-19 patient platelet-specific transcriptional characteristics can be used to identify potential drugs for therapeutic repurposing

**Fig. 7:**
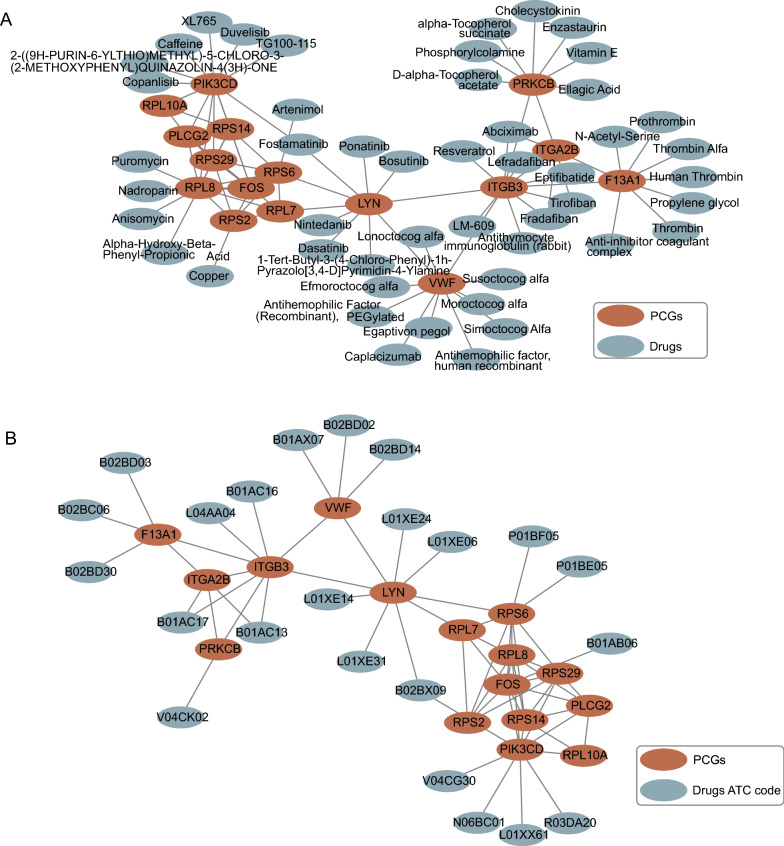
Gene-drug network. **A** Interaction network of drugs and their target mRNAs. **B** Interaction network of approved drug ATC classifications and their target mRNAs. ATC, Anatomical Therapeutic Chemical

We used core mRNAs to identify potential drugs and antiviral agents to analyze their interactions with different drugs and constructed a gene-drug network (Fig. 7A). We identified 54 drugs that acted against the 16 key regulators, implying that these key regulators have been widely used as drug targets to treat different diseases, and genes that have no connection with drugs in the network may be potential drug targets. In addition, we replaced these drugs with their ATC classification and constructed a gene-drug ATC coding network (Fig. 7B) to gain further information about the drugs. Most of the drugs were used for blood and blood-forming organs and acted as antineoplastic and immunomodulating agents. Since these genes were identified as potential drug targets, we inputted these genes into the CMAP database to predict the candidate chemicals. The top 10 drugs associated with the downregulated expression of mRNAs in COVID-19 patients are shown in Table 2, amongst which gabexate, valproic acid, estradiol, ketorolac and pronetalol have already been reported in previous studies. As these signature drugs were detected to target the key regulators, these drugs may represent potential drugs for COVID-19 and stimulate platelet activation.

**Table 2:** Suggested top chemicals for the Platelet changes caused by SARS-CoV-2 infection

Chemical	Dose (μM)	Cell	Score	Up	Down	References
Gabexate	10	PC3	1	0.812	− 0.237	Yes
Phthalylsulfathiazole	10	MCF7	0.874	0.720	− 0.198	–
Bisacodyl	11	HL60	0.868	0.694	− 0.217	–
Boldine	12	PC3	0.858	0.567	− 0.333	–
Valproic acid	500	HL60	0.842	0.683	− 0.200	Yes
Estradiol	100	MCF7	0.838	0.679	− 0.201	Yes
Ketorolac	11	MCF7	0.833	0.642	− 0.233	Yes
Dacarbazine	22	HL60	0.829	0.754	− 0.116	–
Pronetalol	15	MCF7	0.825	0.675	− 0.191	Yes
GlycopyrroniumBromide	10	PC3	0.820	0.667	− 0.193	–

## Discussion

Since the outbreak of COVID-19, an increasing number of studies have reported the hyperactivity of activated platelets in COVID-19 patients [26, 42, 43], and their role in immunothrombosis induced by SARS-CoV-2 [20, 44]. Emerging evidence has shown that biomarkers of platelet activation were significantly increased in COVID-19 patients and were associated with thrombosis and increased mortality in hospitalized COVID-19 patients, including CD40 ligand (sCD40L), P-selectin, the metabolite of thromboxane A2, thromboxane B2 (TxB2) [45, 46]. Thus, it remains an important research priority to clarify the mechanisms underlying the interaction between platelets and SARS-CoV-2 to provide potential treatment strategies for immunothrombosis during COVID-19.

We performed RNA-seq on RNA isolated from platelets obtained from 10 COVID-19 patients and eight healthy donors in the present study. We found that SARS-CoV-2 significantly altered the coding and non-coding transcriptional landscape (Fig. 2 and Additional file 1: Figure S1). However, the platelets and coagulation indicators of COVID-19 patients were within the normal range or only slightly exceeded the normal range (Fig. 1 and Table 1). We further performed functional and tissue enrichment analysis and discovered that SARS-CoV-2 changed the function of the platelets, promoted thrombosis and affected their energy metabolism. However, the activation and hyperresponsiveness of platelets in patients with infectious diseases are known to be caused by numerous factors. For example, during excessive inflammation, the accumulation of proinflammatory cytokines and chemokines has been found to activate platelets indirectly [58]. Viruses, such as DENV, influenza virus [47] and human immunodeficiency virus [48], have also been identified to activate platelets directly. Therefore, the current study conducted further analysis to explore how SARS-CoV-2 may activate and regulate platelets.

We constructed a dysregulated gene co-expression network based on differentially expressed mRNAs and lncRNAs and identified four key subnetworks in the circulating platelets during SARS-CoV-2 infection. The four key subnetworks were found to be involved in ‘coronavirus disease-COVID-19’, ‘platelet activation’ and ‘immune response pathways’. These results indicated that the four key subnetworks might contain the key factor that SARS-CoV-2 infection regulates to promote platelet activation. We subsequently mapped these mRNAs to ‘coronavirus disease-COVID-19’ and ‘platelet activation pathways’ and identified 16 key candidate regulators (Fig. 5A). In previous studies, Palma et al. found that PI3Kδ inhibition could be used as a potential therapeutic target for COVID-19 to reduce inflammation and patient death [49]. A single-center cross-sectional study report showed that VWF activity and antigen levels continued to increase as the disease progressed [50], which was associated with the poor prognosis of COVID-19 patients [51]. Another comparative study discovered that integrin and integrin signaling genes, including integrin subunit β 3 and integrin subunit α 2b, were highly expressed in lung samples and were suggested to provide SARS-CoV-2 with a more effective competitive advantage for invading lung cells [52]. The previous published experimental work provides further evidence for the core role of the 16 key candidate regulators in the regulation of platelets following SARS-CoV-2 infection.

Notably, we observed a significant overlap between the co-expression relationship and PPI among the 16 key candidate regulators. Thus, we combined the results obtained from the enrichment analyses, identification of key subnetworks and regulators, the PPI network and reported studies to hypothesize that a significant alteration in the VWF/GP Ib-IX-V axis may be strongly associated with platelet aggregation and immunothrombosis. It is well-established that the entry of SARS-CoV-2 into cells relies on angiotensin-converting enzyme 2 (ACE2) and transmembrane protease serine 2 (TMPRSS2) for viral spike protein initiation, and this receptor is highly expressed by nasopharyngeal airway and alveolar epithelial cells, vascular endothelial cells and lung macrophages [53]. However, an increased number of studies have suggested that platelets do not express ACE2 and TMPRSS2 and the activation and regulation of platelets by SARS-CoV-2 seems to occur independently of ACE and TMPRSS2 [26, 42, 54]. VWF is a multimeric glycoprotein in the plasma, which plays a vital role in hemostasis and thrombosis by mediating the adhesion of platelets to damaged and activated blood vessels [55]. During infection, inflammation may regulate the formation of VWF-platelet thrombi in large arteries and small blood vessels by increasing VWF levels, enhancing VWF responsiveness and regulating the level and activity of regulatory molecules in the circulation [56]. This explains the significant changes in platelet VWF/GP Ib-IX-V levels in patients with COVID-19 in the present study, and further indicates that the cascade initiated by the VWF/GP Ib-IX-V complex may play a pivotal role in the activation and regulation of platelets by SARS-CoV-2 infection.

Furthermore, the course of COVID-19 develops rapidly and can lead to severe and fatal complications, such as acute myocardial injury and chronic damage to the cardiovascular system [40, 41]. To facilitate the stratification of high-risk patients, reliable biomarkers related to disease progression are urgently required in the clinic. In the present study, four risk regulators (UBTF, POLR2L, YBX1 and YPEL2) were identified to be associated with COVID-19 severity. Therefore, these four regulators may represent potential biomarkers for the clinical management of COVID-19 patients.

Immunothrombosis is strongly associated with the progression of COVID-19 disease. Timely and accurate anticoagulation and vigilant monitoring of thrombotic complications are key to COVID-19 patient management [57]. The current study identified 54 drugs targeting the 16 key regulators and five out of the top 10 drugs had been previously reported in the literature. Thus, they may represent potential drugs for the treatment of COVID-19-associated thrombosis.

## Conclusions

The findings of the present study provided novel evidence to suggest that SARS-CoV-2 may significantly alter the coding and non-coding transcriptional landscape of platelets and subsequently alter their function. The altered transcriptional landscape and the identification of the critical subnetwork and risk regulators of platelets provided novel insights into the molecular mechanisms of immunothrombosis in COVID-19 progression, which paves the way for developing new therapeutic strategies for preventing COVID-19-associated thrombosis and improving the clinical outcome of COVID-19 patients.

## Methods

### Subjects and specimen collection

The current study was approved by the Institutional Review Board (IRB) of The Second Affiliated Hospital of Wenzhou Medical University (IRB# LCKY2020-09 and LCKY2020-222), and written informed consent was obtained from each participant prior to the commencement of the study. A total of 10 patients with COVID-19 were recruited from The Second Affiliated Hospital of Wenzhou Medical University between 6 and 16 February 2020, and the control group consisting of eight donors were recruited between 4 May and 15 August 2020. COVID-19 cases were confirmed by detecting SARS-CoV-2 RNA using reverse transcription-quantitative PCR. All donors of the control group underwent preliminary screening and the following exclusion criteria were applied: (i) Use of antiplatelet drugs; (ii) recent infections; and (iii) diagnosis of myelosuppressive diseases. K2-EDTA-anticoagulated whole blood was collected from hospitalized COVID-19 patients before antiplatelet or anticoagulation therapy. In addition, the blood count and C reactive protein (CRP) were detected by Sysmex XE 5000 hematology analyzer (Sysmex Corporation, Kobe, Japan), the coagulation parameters were detected using an automation coagulator STA-R Evolution (Stago, NJ, USA), and biochemical Indicators were measured using Cobas 6000 c501 (Roche Diagnostics, Rotkreuz, Switzerland). The clinical data of all participants, including age, sex, dietary history, medication use and laboratory parameters, were collected and are shown in Table 1.

Another platelet RNA-seq cohort of 10 COVID-19 patients (including six non-ICU and four ICU COVID-19 patients) was retrieved from NCBI short-read archives under PRJNA634489 [58].

### Platelet isolation and sequencing

The samples of all hospitalized patients were taken from the first morning after the patients were admitted to the hospital. The same standardized operating procedure was used to collect the whole blood from COVID-19 patients and healthy donors. Platelet parameters were measured using a Sysmex XE 5000 hematology analyzer (Sysmex Corporation, Kobe, Japan). Platelets were isolated from the whole blood within 12 h and leukocyte-removed platelets were obtained using the previously described method [59–61]. Briefly, whole blood collected in the 5 ml EDTA-coated vacuum tube was centrifuged at 120×*g* for 20 min to separate the platelet-rich plasma (PRP) from the nucleated blood cells. The PRP was then centrifuged using a 20-min 360×*g* step to pellet the platelets. Subsequently, 9/10th of the upper PRP was carefully removed and the platelet pellet was collected and resuspended in RNAlater, then frozen at − 80 °C. The counts and the purity of the separated platelets were confirmed using flow cytometry.

For next-generation RNA-seq, total RNA was isolated from 1 × 10^9^ separated platelets using TRIzol® reagent (Invitrogen; Thermo Fisher Scientific, Inc., Waltham, MA, USA), followed by DNase treatment. The quantity and quality of total RNA were determined and used for RNA library construction.

### RNA-seq analysis

Quality control of raw RNA-seq data was conducted using Trimmomatic (v0.39) [62], including read trimming and quality filtering. Clean RNA-seq reads were mapped to the human reference genome (hg19) that included GENCODE v29 gene annotation using the HISAT2 program (v2.2.1, with default parameters). Alignment files were processed and the final BAM files were obtained using SAMtools (V1.7) [63]. The number of reads mapping each human gene (as annotated in the GENCODE v29 gene annotation) was counted using HTSeq v0.11.2 [64]. Differential expression analysis was conducted using the DESeq2 package [65], and statistical significances in expression between COVID-19 patients and healthy individuals were determined using a false discovery rate-adjusted P-value < 0.05 and a log_2_fold change (FC) > 1.0.

### Gene enrichment analyses

Functional enrichment analyses of differentially expressed RNAs, including Gene Ontology (GO) functional term and Kyoto Encyclopedia of Genes and Genomes (KEGG) signaling pathway enrichment analyses, and tissue and cell-specific enrichment analysis [66], were performed and visualized using the Metascape gene enrichment analysis tool (https://metascape.org) [66] and R package ‘clusterProfiler’ [67]. Enrichment analysis of differentially expressed RNAs in virus perturbations datasets obtained from the Gene Expression Omnibus (GEO) database was conducted using Enrichr (https://amp.pharm.mssm.edu/Enrichr) [68].

### Co-expression network analysis

The Pearson’s correlation coefficient (PCC) was calculated to measure the linear expression correlation between each pair of differentially expressed RNAs. A dysregulated gene co-expression network (dysGCN), which was composed of differentially expressed protein-coding genes (PCGs) and long non-coding RNAs (lncRNAs) as points and a PCC of > 0.8 as edges, was then constructed and visualized using Cytoscape (http://www.cytoscape.org). The densely connected and bipartite network modules were identified using Molecular Complex Detection (http://apps.cytoscape.org/apps/mcodeplugin) of Cytoscape software [69].

### Drug prediction analysis

Proteins are the direct targets of drugs and the bridge connecting genes and targeted drugs; thus, we mapped the RNA to protein using the UniProt database (https://www.uniprot.org). The UniProt ID was input into the DrugBank database (https://go.drugbank.com) to identify drugs related to the proteins. To obtain more information about the drugs, we also recorded the Anatomical Therapeutic Chemical (ATC) classification of the drugs and constructed a gene-drug ATC coding network. Since these genes can be used as disease targets, some candidate chemicals have been identified to predict the chemicals that target these genes and are related to the treatment of COVID-19 and bleeding. The Connectivity Map (CMAP) database [19] uses gene-expression signatures to predict small molecular compounds for a specific disease. Upregulated and downregulated genes were subsequently uploaded to the CMAP database and small molecule drugs that may treat COVID-19 and bleeding were searched for.

### Real-Time Quantitative PCR (RT-qPCR)

A total of 5 patients with pneumonia and 6 control donors were recruited from The Second Affiliated Hospital of Wenzhou Medical University on 17 December 2021. The samples of patients were taken from the first morning after the patients were admitted to the hospital and the samples of control donors were collected on the morning of the physical examination. Platelet total RNA from all samples was extracted according to the method described above within 12 h. Total RNA was reversely transcribed into cDNA using the reverse transcriptase reaction kit (vazyme). DNA removal was conducted at 42 °C for 2 min with the addition of 4ul 4XgDNA wiper mix and 12ul RNA, and then reverse transcription reaction was conducted at 50 °C for 15 min and 85 °C for 5 s with the addition of 4ul 5X hiscript qRT supermix. RT-qPCR was carried out on a Bio-Rad C1000 quantitative PCR instrument with the SYBR Select mastermix (Applied Biosystems) kits. The amplification conditions were: 2 min at 50 °C, and 2 min at 95 °C; 15 s at 95 °C, and 1 min at 60 °C (40cycles). Glyceraldehyde-3-phosphate dehydrogenase (GAPDH) was used as the internal reference for the mRNAs. The ΔCq was used to calculate the expression of the target gene in the patient's group and the control group. The primer sequences were shown in Additional file 6: Table S2. The primers were synthesized by Sangon Biotech (Shanghai, China).

## Supplementary Information

**Additional file 1: Figure S1.** The imaging of all patients.

**Additional file 2: Figure S2.** Hierarchical clustering of transcriptome-wide RNA expression. Hierarchical clustering of samples according to global gene expression segregates SARS-CoV-2-infected patients (red numbers) from healthy donors (gray numbers). SARS-CoV-2, severe acute respiratory syndrome coronavirus 2.

**Additional file 3: Figure S3.** Functional characterization of key regulators in circulating platelets during SARS-CoV-2 infection. (A) Tissue and cell-specific enrichment analysis. (B) Disease enrichment analysis. (C) Enrichment analysis of differentially expressed RNAs in viral perturbations datasets from the Gene Expression Omnibus database. SARS-CoV-2, severe acute respiratory syndrome coronavirus 2.

**Additional file 4: Figure S4.** Boxplots showing expression levels of four key regulators using RT-qPCR.

**Additional file 5: Table S1.** Lists of differentially expressed RNAs between COVID-19 patients and healthy donors.

**Additional file 6: Table S2.** Primers sequences for four candidate mRNAs and reference mRNA.

## Data Availability

The datasets used and/or analyzed during the present study are available from the corresponding author on reasonable request.

## Abbreviations

COVID-19: Coronavirus disease 2019
SARS-CoV-2: Severe acute respiratory syndrome coronavirus 2
ICUs: Intensive care units
DENV: Dengue virus
CRP: C reactive protein
GEO: Gene Expression Omnibus
PCC: Pearson’s correlation coefficient
CMAP: Connectivity Map
PT: Prothrombin time
aPTT: Activated partial thromboplastin time
VWF: Von Willebrand factor
OMIM: Online Mendelian Inheritance in Man
ACE2: Angiotensin-converting enzyme 2
TMPRSS2: Transmembrane protease serine 2
sCD40L: CD40 ligand

## References

[1] Wan Y, Shang J, Graham R, Baric RS, Li F (2020). Receptor Recognition by the Novel Coronavirus from Wuhan: an analysis based on decade-long structural studies of sars coronavirus. J Virol.

[2] Tncpere T (2020). The Epidemiological characteristics of an outbreak of 2019 novel Coronavirus diseases (COVID-19)—China, 2020. CCDCW.

[3] Zhou P, Yang XL, Wang XG, Hu B, Zhang L, Zhang W (2020). A pneumonia outbreak associated with a new coronavirus of probable bat origin. Nature.

[4] Wu ZMJ (2020). Characteristics of and Important Lessons From the Coronavirus Disease 2019 (COVID-19) Outbreak in China: Summary of a Report of 72 314 Cases From the Chinese Center for Disease Control and Prevention. JAMA.

[5] Ruan Q, Yang K, Wang W, Jiang L, Song J (2020). Clinical predictors of mortality due to COVID-19 based on an analysis of data of 150 patients from Wuhan. China Intensive Care Med.

[6] Tian S, Hu N, Lou J, Chen K, Kang X, Xiang Z (2020). Characteristics of COVID-19 infection in Beijing. J Infect.

[7] Scavone CBS, Bertini M, Sportiello L, Rafaniello C, Zoccoli A, Berrino L, Racagni G, Rossi F, Capuano A (2020). Current pharmacological treatments for COVID-19: What's next?. Br J Pharmacol.

[8] Zhao Z, Bourne PE (2020). Structural Insights into the Binding Modes of Viral RNA-Dependent RNA polymerases using a function-site interaction fingerprint method for RNA Virus Drug Discovery. J Proteome Res.

[9] Shi R, Lai C, Teboul JL, Dres M, Moretto F, De Vita N (2021). COVID-19 ARDS is characterized by higher extravascular lung water than non-COVID-19 ARDS: the PiCCOVID study. Crit Care.

[10] Matsuyama T, Kubli SP, Yoshinaga SK, Pfeffer K, Mak TW (2020). An aberrant STAT pathway is central to COVID-19. Cell Death Differ.

[11] Sriram K, Insel PA (2021). Inflammation and thrombosis in COVID-19 pathophysiology: proteinase-activated and purinergic receptors as drivers and candidate therapeutic targets. Physiol Rev.

[12] Ackermann M, Verleden SE, Kuehnel M, Haverich A, Welte T, Laenger F (2020). Pulmonary vascular endothelialitis, thrombosis, and angiogenesis in covid-19. N Engl J Med.

[13] Iba T, Levy JH, Levi M, Connors JM, Thachil J (2020). Coagulopathy of coronavirus disease 2019. Crit Care Med.

[14] Chiumello D, Busana M, Coppola S, Romitti F, Formenti P, Bonifazi M (2020). Physiological and quantitative CT-scan characterization of COVID-19 and typical ARDS: a matched cohort study. Intensive Care Med.

[15] Klok FA, Kruip M, van der Meer NJM, Arbous MS, Gommers D, Kant KM (2020). Confirmation of the high cumulative incidence of thrombotic complications in critically ill ICU patients with COVID-19: An updated analysis. Thromb Res.

[16] Helms J, Tacquard C, Severac F, Leonard-Lorant I, Ohana M, Delabranche X (2020). High risk of thrombosis in patients with severe SARS-CoV-2 infection: a multicenter prospective cohort study. Intensive Care Med.

[17] Goldenberg NA, Sochet A, Albisetti M, Biss T, Bonduel M, Jaffray J (2020). Consensus-based clinical recommendations and research priorities for anticoagulant thromboprophylaxis in children hospitalized for COVID-19-related illness. J Thromb Haemost.

[18] Feldstein LR, Rose EB, Horwitz SM, Collins JP, Newhams MM, Son MBF (2020). Multisystem Inflammatory Syndrome in US Children and Adolescents. N Engl J Med.

[19] Tang N, Li D, Wang X, Sun Z (2020). Abnormal coagulation parameters are associated with poor prognosis in patients with novel coronavirus pneumonia. J Thromb Haemost.

[20] McFadyen JD, Stevens H, Peter K (2020). The Emerging Threat of (Micro)Thrombosis in COVID-19 and Its Therapeutic Implications. Circ Res.

[21] Lax SF, Skok K, Zechner P, Kessler HH, Kaufmann N, Koelblinger C (2020). Pulmonary Arterial Thrombosis in COVID-19 with fatal outcome : results from a prospective, single-center. Clinicopathologic Case Series Ann Intern Med.

[22] Wichmann D, Sperhake JP, Lutgehetmann M, Steurer S, Edler C, Heinemann A (2020). Autopsy findings and venous thromboembolism in patients with COVID-19: a prospective cohort study. Ann Intern Med.

[23] Rapkiewicz AV, Mai X, Carsons SE, Pittaluga S, Kleiner DE, Berger JS (2020). Megakaryocytes and platelet-fibrin thrombi characterize multi-organ thrombosis at autopsy in COVID-19: A case series. EClinicalMedicine..

[24] Shen S, Zhang J, Fang Y, Lu S, Wu J, Zheng X (2021). SARS-CoV-2 interacts with platelets and megakaryocytes via ACE2-independent mechanism. J Hematol Oncol.

[25] Aid M, Busman-Sahay K, Vidal SJ, Maliga Z, Bondoc S, Starke C (2020). Vascular disease and thrombosis in SARS-CoV-2-infected rhesus macaques. Cell.

[26] Manne BKDF, Middleton EA, Portier I, Rowley JW, Stubben C, Petrey AC, Tolley ND, Guo L, Cody M, Weyrich AS, Yost CC, Rondina MT, Campbell RA (2020). Platelet gene expression and function in patients with COVID-19. Blood.

[27] Junt TSH, Chen Z, Massberg S, Goerge T, Krueger A, Wagner DD, Graf T, Italiano JE, Shivdasani RA, von Andrian UH (2007). Dynamic visualization of thrombopoiesis within bone marrow. Science.

[28] Zm R (2002). Platelets in atherothrombosis. Nat Med.

[29] Petrey AC, Obery DR, Kessler SP, Zawerton A, Flamion B, de la Motte CA (2019). Platelet hyaluronidase-2 regulates the early stages of inflammatory disease in colitis. Blood.

[30] Li Guo MTR (2019). The era of thromboinflammation: platelets are dynamic sensors and effector cells during infectious diseases. Front Immunol.

[31] Rondina MT, Weyrich AS (2015). Regulation of the genetic code in megakaryocytes and platelets. J Thromb Haemost.

[32] Rowley JW, Oler AJ, Tolley ND, Hunter BN, Low EN, Nix DA (2011). Genome-wide RNA-seq analysis of human and mouse platelet transcriptomes. Blood.

[33] Guan WJ, Ni ZY, Hu Y, Liang WH, Ou CQ, He JX (2020). Clinical characteristics of coronavirus disease 2019 in China. N Engl J Med.

[34] Dennis JM, Mateen BA, Sonabend R, Thomas NJ, Patel KA, Hattersley AT (2021). Type 2 Diabetes and COVID-19-related mortality in the critical care setting: a national cohort study in England, March-July 2020. Diabetes Care.

[35] Richardson S, Hirsch JS, Narasimhan M, Crawford JM, McGinn T, Davidson KW (2020). Presenting characteristics, comorbidities, and outcomes among 5700 patients hospitalized With COVID-19 in the New York City Area. JAMA.

[36] Li S, Jiang L, Li X, Lin F, Wang Y, Li B (2020). Clinical and pathological investigation of patients with severe COVID-19. JCI Insight..

[37] Wu C, Chen X, Cai Y, Xia J, Zhou X, Xu S (2020). Risk factors associated with acute respiratory distress syndrome and death in patients with coronavirus disease 2019 Pneumonia in Wuhan. China JAMA Intern Med.

[38] Chen G, Wu D, Guo W, Cao Y, Huang D, Wang H (2020). Clinical and immunological features of severe and moderate coronavirus disease 2019. J Clin Invest.

[39] Iadecola C, Anrather J, Kamel H (2020). Effects of COVID-19 on the Nervous System. Cell.

[40] Zheng YY, Ma YT, Zhang JY, Xie X (2020). COVID-19 and the cardiovascular system. Nat Rev Cardiol.

[41] Siddiqi HK, Libby P, Ridker PM (2021). COVID-19 - A vascular disease. Trends Cardiovasc Med.

[42] Zaid Y, Puhm F, Allaeys I, Naya A, Oudghiri M, Khalki L (2020). Platelets Can Associate with SARS-Cov-2 RNA and Are Hyperactivated in COVID-19. Circ Res.

[43] Koupenova M, Freedman JE (2020). Platelets and COVID-19: Inflammation, Hyperactivation and Additional Questions. Circ Res.

[44] Lippi G, Sanchis-Gomar F, Favaloro EJ, Lavie CJ, Henry BM (2021). Coronavirus Disease 2019-Associated Coagulopathy. Mayo Clin Proc.

[45] Barrett TJ, Lee AH, Xia Y, Lin LH, Black M, Cotzia P (2020). Platelet and vascular biomarkers associate with thrombosis and death in coronavirus disease. Circ Res.

[46] Hottz ED A-QI, Palhinha L, Teixeira L, Barreto EA, Pão CRR, Righy C, Franco S, Souza TML, Kurtz P, Bozza FA, Bozza PT. Platelet activation and platelet-monocyte aggregates formation trigger tissue factor expression in severe COVID-19 patients. Blood. 2020;136(11):1330–41. 10.1182/blood.2020007252 PMC7483437 32678428

[47] Campbell RA, Schwertz H, Hottz ED, Rowley JW, Manne BK, Washington AV (2019). Human megakaryocytes possess intrinsic antiviral immunity through regulated induction of IFITM3. Blood.

[48] Youssefian T, Drouin A, Masse JM, Guichard J, Cramer EM (2002). Host defense role of platelets: engulfment of HIV and Staphylococcus aureus occurs in a specific subcellular compartment and is enhanced by platelet activation. Blood.

[49] Palma G, Pasqua T, Silvestri G, Rocca C, Gualtieri P, Barbieri A (2020). PI3Kdelta Inhibition as a Potential Therapeutic Target in COVID-19. Front Immunol.

[50] Goshua G, Pine AB, Meizlish ML, Chang CH, Zhang H, Bahel P (2020). Endotheliopathy in COVID-19-associated coagulopathy: evidence from a single-centre, cross-sectional study. The Lancet Haematology.

[51] Wibowo A, Pranata R, Lim MA, Akbar MR, Martha JW (2021). Endotheliopathy Marked by High von Willebrand Factor (vWF) Antigen in COVID-19 is Associated with Poor Outcome: A Systematic Review and Meta-analysis. Int J Infect Dis.

[52] Islam A, Khan MA, Ahmed R, Hossain MS, Kabir SMT, Islam MS (2021). Transcriptome of nasopharyngeal samples from COVID-19 patients and a comparative analysis with other SARS-CoV-2 infection models reveal disparate host responses against SARS-CoV-2. J Transl Med.

[53] Xu H, Zhong L, Deng J, Peng J, Dan H, Zeng X (2020). High expression of ACE2 receptor of 2019-nCoV on the epithelial cells of oral mucosa. Int J Oral Sci.

[54] Middleton EA, Rowley JW, Campbell RA, Grissom CK, Brown SM, Beesley SJ (2019). Sepsis alters the transcriptional and translational landscape of human and murine platelets. Blood.

[55] Je S (1998). Biochemistry and genetics of von Willebrand factor. Annu Rev Biochem.

[56] Chen JCD (2018). Inflammation, von Willebrand factor, and ADAMTS13. Blood.

[57] Shaw RJ, Bradbury C, Abrams ST, Wang G, Toh CH (2021). COVID-19 and immunothrombosis: emerging understanding and clinical management. Br J Haematol.

[58] Manne BK, Denorme F, Middleton EA, Portier I, Rowley JW, Stubben C (2020). Platelet gene expression and function in patients with COVID-19. Blood.

[59] Best MG, Veld S, Sol N, Wurdinger T (2019). RNA sequencing and swarm intelligence-enhanced classification algorithm development for blood-based disease diagnostics using spliced blood platelet RNA. Nat Protoc.

[60] Best MG, Sol N, Veld S, Vancura A, Muller M, Niemeijer AN (2017). Swarm intelligence-enhanced detection of non-small-cell lung cancer using tumor-educated platelets. Cancer Cell.

[61] Campbell RA, Franks Z, Bhatnagar A, Rowley JW, Manne BK, Supiano MA (2018). Granzyme A in human platelets regulates the synthesis of proinflammatory cytokines by monocytes in aging. J Immunol.

[62] Bolger AM, Lohse M, Usadel B (2014). Trimmomatic: a flexible trimmer for Illumina sequence data. Bioinformatics.

[63] Li H, Handsaker B, Wysoker A, Fennell T, Ruan J, Homer N (2009). The Sequence Alignment/Map format and SAMtools. Bioinformatics.

[64] Anders S, Pyl PT, Huber W (2015). HTSeq–a Python framework to work with high-throughput sequencing data. Bioinformatics.

[65] Love MI, Huber W, Anders S (2014). Moderated estimation of fold change and dispersion for RNA-seq data with DESeq2. Genome Biol.

[66] Zhou Y, Zhou B, Pache L, Chang M, Khodabakhshi AH, Tanaseichuk O (2019). Metascape provides a biologist-oriented resource for the analysis of systems-level datasets. Nat Commun.

[67] Yu G, Wang LG, Han Y, He QY (2012). clusterProfiler: an R package for comparing biological themes among gene clusters. OMICS.

[68] Kuleshov MV, Jones MR, Rouillard AD, Fernandez NF, Duan Q, Wang Z (2016). Enrichr: a comprehensive gene set enrichment analysis web server 2016 update. Nucleic Acids Res.

[69] Bader GD, Hogue CW (2003). An automated method for finding molecular complexes in large protein interaction networks. BMC Bioinformatics.

