# Supplementary figures and images for: Transcriptional landscape of circulating platelets from patients with COVID-19 reveals key subnetworks and regulators underlying SARS-CoV-2 infection: implications for immunothrombosis
Source: Cell Biosci. 2022 Feb 9;12:15. doi: 10.1186/s13578-022-00750-5 (PMC8827164; doi:10.1186/s13578-022-00750-5)

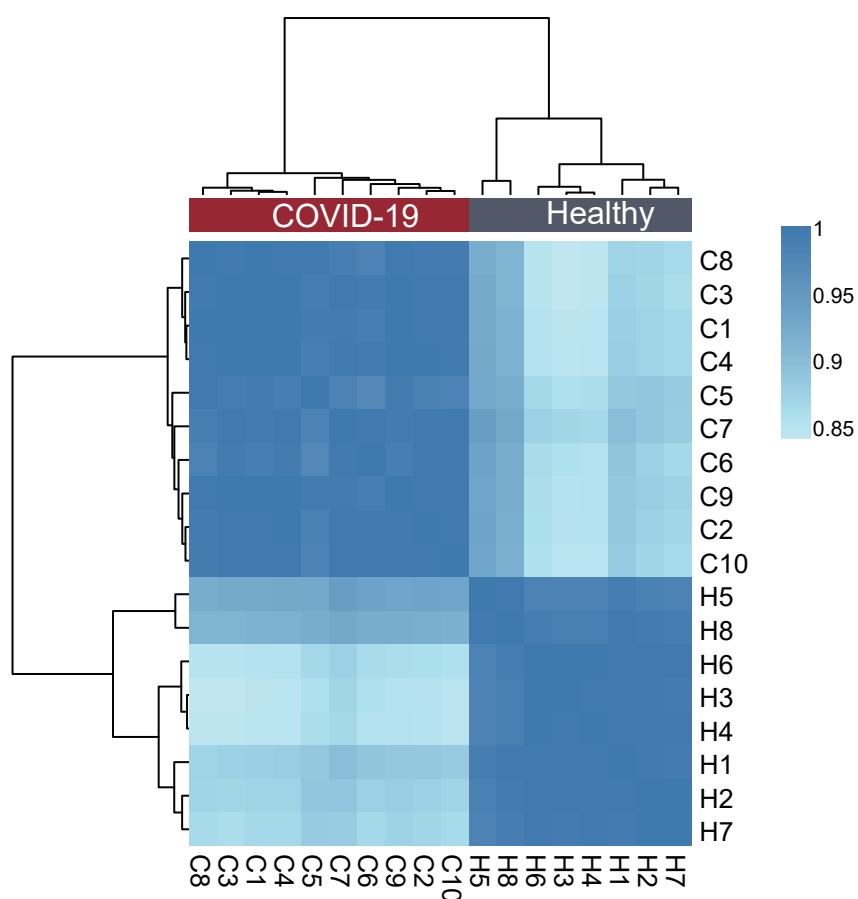

Supplement: Supplementary file 1 — Additional file 1: Figure S1. The imaging of all patients. [file 13578_2022_750_MOESM1_ESM.pdf]

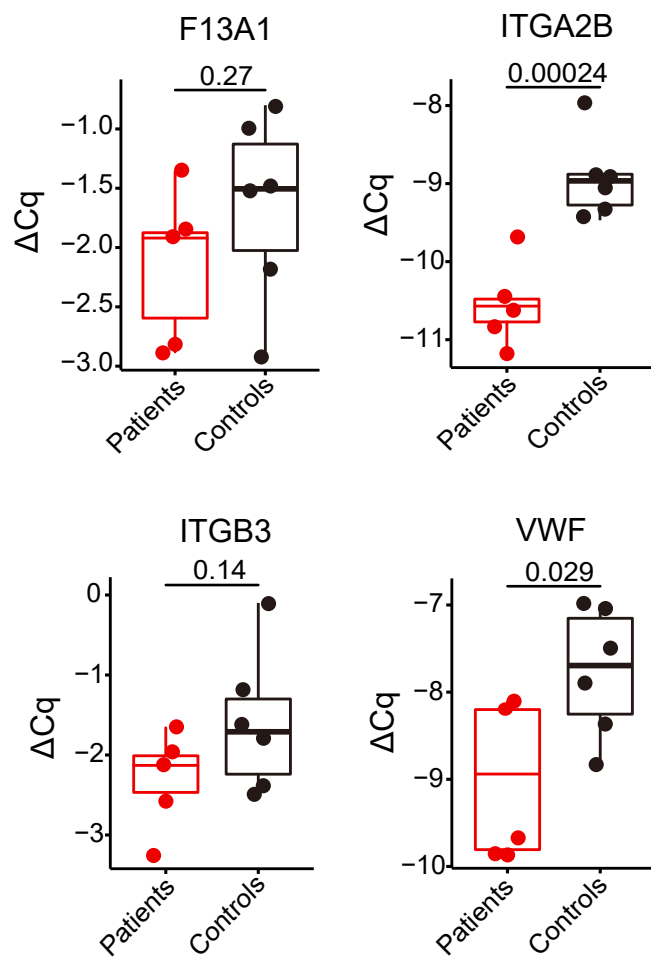

Supplement: Supplementary file 4 — Additional file 4: Figure S4. Boxplots showing expression levels of four key regulators using RT-qPCR. [file 13578_2022_750_MOESM4_ESM.pdf]
