# Supplementary material for: Transcriptional landscape of circulating platelets from patients with COVID-19 reveals key subnetworks and regulators underlying SARS-CoV-2 infection: implications for immunothrombosis
Source: Cell Biosci. 2022 Feb 9;12:15. doi: 10.1186/s13578-022-00750-5 (PMC8827164; doi:10.1186/s13578-022-00750-5)
Supplement: Supplementary file 2 — Additional file 2: Figure S2. Hierarchical clustering of transcriptome-wide RNA expression. Hierarchical clustering of samples according to global gene expression segregates SARS-CoV-2-infected patients (red numbers) from healthy donors (gray numbers). SARS-CoV-2, severe acute respiratory syndrome coronavirus 2. [file 13578_2022_750_MOESM2_ESM.pdf]

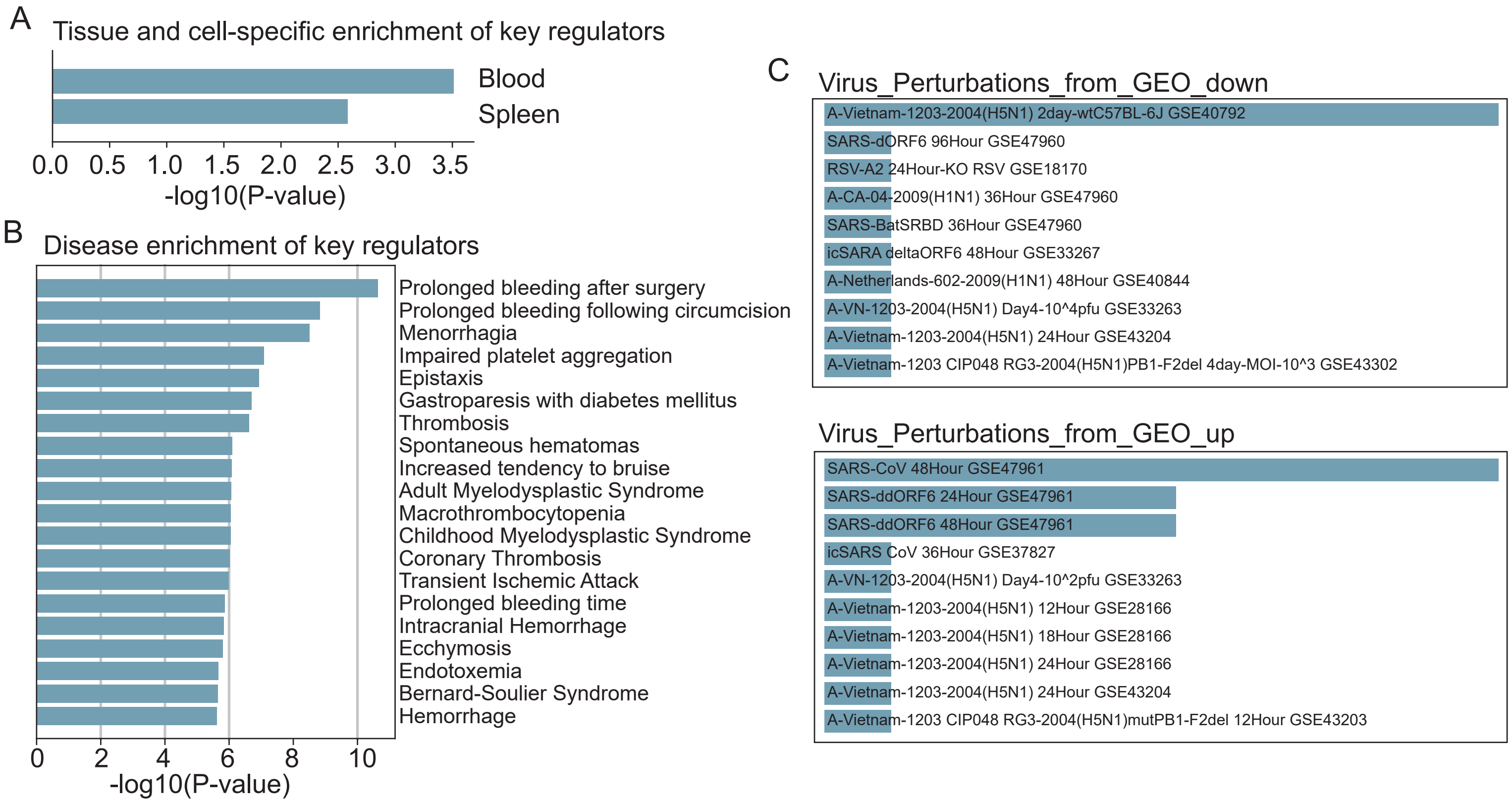
A
Tissue and cell-specific enrichment of key regulators
Blood
Spleen
0.0 0.5 1.0 1.5 2.0 2.5 3.0 3.5
-log10(P-value)
B
Disease enrichment of key regulators
Prolonged bleeding after surgery
Prolonged bleeding following circumcision
Menorrhagia
Impaired platelet aggregation
Epistaxis
Gastroparesis with diabetes mellitus
Thrombosis
Spontaneous hematomas
Increased tendency to bruise
Adult Myelodysplastic Syndrome
Macrothrombocytopenia
Childhood Myelodysplastic Syndrome
Coronary Thrombosis
Transient Ischemic Attack
Prolonged bleeding time
Intracranial Hemorrhage
Ecchymosis
Endotoxemia
Bernard-Soulier Syndrome
Hemorrhage
0 2 4 6 8 10
-log10(P-value)
C
Virus_Perturbations_from_GEO_down
A-Vietnam-1203-2004(H5N1) 2day-wtC57BL-6J GSE40792
SARS-dORF6 96Hour GSE47960
RSV-A2 24Hour-KO RSV GSE18170
A-CA-04-2009(H1N1) 36Hour GSE47960
SARS-BatSRBD 36Hour GSE47960
icSARA deltaORF6 48Hour GSE33267
A-Netherlands-602-2009(H1N1) 48Hour GSE40844
A-VN-1203-2004(H5N1) Day4-10^4pfu GSE33263
A-Vietnam-1203-2004(H5N1) 24Hour GSE43204
A-Vietnam-1203 CIP048 RG3-2004(H5N1)PB1-F2del 4day-MOI-10^3 GSE43302
Virus_Perturbations_from_GEO_up
SARS-CoV 48Hour GSE47961
SARS-ddORF6 24Hour GSE47961
SARS-ddORF6 48Hour GSE47961
icSARS CoV 36Hour GSE37827
A-VN-1203-2004(H5N1) Day4-10^2pfu GSE33263
A-Vietnam-1203-2004(H5N1) 12Hour GSE28166
A-Vietnam-1203-2004(H5N1) 18Hour GSE28166
A-Vietnam-1203-2004(H5N1) 24Hour GSE28166
A-Vietnam-1203-2004(H5N1) 24Hour GSE43204
A-Vietnam-1203 CIP048 RG3-2004(H5N1)mutPB1-F2del 12Hour GSE43203
