# Supplementary material for: Transcriptional landscape of circulating platelets from patients with COVID-19 reveals key subnetworks and regulators underlying SARS-CoV-2 infection: implications for immunothrombosis
Source: Cell Biosci. 2022 Feb 9;12:15. doi: 10.1186/s13578-022-00750-5 (PMC8827164; doi:10.1186/s13578-022-00750-5)
Supplement: Supplementary file 3 — Additional file 3: Figure S3. Functional characterization of key regulators in circulating platelets during SARS-CoV-2 infection. (A) Tissue and cell-specific enrichment analysis. (B) Disease enrichment analysis. (C) Enrichment analysis of differentially expressed RNAs in viral perturbations datasets from the Gene Expression Omnibus database. SARS-CoV-2, severe acute respiratory syndrome coronavirus 2. [file 13578_2022_750_MOESM3_ESM.pdf]

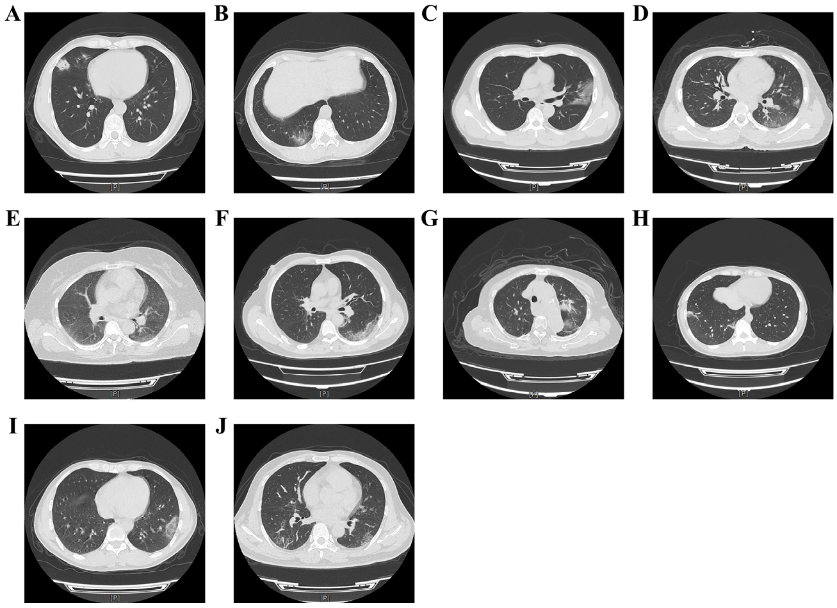

Figure S3 Imaging characteristics of hospitalized patients with COVID-19, A-J stands for C1-C10 respectively.
