# Supplementary material for: Transcriptional landscape of circulating platelets from patients with COVID-19 reveals key subnetworks and regulators underlying SARS-CoV-2 infection: implications for immunothrombosis
Source: Cell Biosci. 2022 Feb 9;12:15. doi: 10.1186/s13578-022-00750-5 (PMC8827164; doi:10.1186/s13578-022-00750-5)
Supplement: Supplementary file 5 — Additional file 5: Table S1. Lists of differentially expressed RNAs between COVID-19 patients and healthy donors. [file 13578_2022_750_MOESM5_ESM.docx]

**Supplementary Table 1 Primers sequences for four candidate mRNAs and reference mRNA**

| Primer | Sequence (5’ to 3’) |
| --- | --- |
| F13A1-F  F13A1-R  ITGB3-F  ITGB3-R  ITGA2B-F  ITGA2B-R  VWF-F  VWF-R | ACAGCCACAACCGTTACACCATC  GGATCAGCACCGCCTCTTTCTTG  AGAAGAGCCAGAGTGTCCCAAGG  TCGGTCGTGGATGGTGATGAGG  GCAATTCTAGCCACCATGAGTCCAG  CTCCTCCTCCTTCCCTTCAGATTCC  TGCGACACCATTGCTGCCTATG  GCCACTCACACTCATACCCGTTC |
